# Moderate physical activity alters the estimation of time, but not space

**DOI:** 10.3389/fpsyg.2022.1004504

**Published:** 2022-10-05

**Authors:** Alessia Tonelli, Claudia Lunghi, Monica Gori

**Affiliations:** ^1^UVIP – Unit for Visually Impaired People, Istituto Italiano di Tecnologia, Genova, Italy; ^2^Laboratoire des Systèmes Perceptifs, Département d’Études Cognitives, École Normale Supérieure, PSL University, CNRS, Paris, France

**Keywords:** temporal perception, spatial perception, physical activity, cycling, estimation

## Abstract

Moderate physical activity can influence cognitive functions and visual cortical activity. However, little is known about the effects of exercise on fundamental perceptual domains, such as spatial and temporal representation. Here we tackled this issue by testing the impact of physical activity on a temporal estimation task in a group of adult volunteers in three different conditions: (1) in a resting condition (baseline), (2) during moderate physical activity (cycling in place – PA), and (3) approximately 15 to 20 min following the physical activity phase, in which participants were seated and returned to a regular heart rate (POST). We show that physical activity specifically impacts time perception, inducing a consistent overestimation for durations in the range of milliseconds. Notably, the effect persisted in the POST session, ruling out the main contribution of either heart rate or cycling rhythmicity. In a control experiment, we found that spatial perception (distance estimation) was unaffected by physical activity, ruling out a major contribution of arousal and fatigue to the observed temporal distortion. We speculate that physical exercise might alter temporal estimation either by up-regulating the dopaminergic system or modulating GABAergic inhibition.

## Introduction

Chronic physical exercise has a beneficial impact on high-level brain functions, improving cognitive processes, memory, and neural plasticity (Collignon et al., 2011; Voss et al., 2013; Baek, 2016). Moreover, its benefits extend to the sensory cortex: moderate physical exercise boosts visual cortical activity and plasticity in both humans (Lunghi and Sale, 2015; Bullock et al., 2017; Cao and Händel, 2019; Lunghi et al., 2019) and animals (Niell and Stryker, 2010; Ayaz et al., 2013; Kaneko and Stryker, 2014; Sansevero et al., 2020). If chronic exercise can lead to long-term benefits, benefits of acute activity have also been shown, especially for acute exercise moderate and limited in time (Brisswalter et al., 2002), while vigorous bouts of exercise can have detrimental effects on cognitive functions.

Related to acute exercise, little is known about the effects of exercise on fundamental aspects of visual perception, such as spatial and temporal representation, which are critical to creating a veridical representation of the external environment.

Temporal estimation is highly context-dependent and can be modulated by several factors, from the physical characteristics of the stimuli (van Wassenhove et al., 2011) to emotional valence (Droit-Volet and Gil, 2009). At the neural level, time perception is related to the dopaminergic and the GABAergic systems and the activity of the supplementary motor area (SMA; Mauk and Buonomano, 2004; Coull et al., 2011). Dopamine release from midbrain centers (substantia nigra pars compacta and ventral tegmental area) modulates the rate of the internal clock (Matell and Meck, 2004; Meck, 2005), while the SMA seems to act as the internal temporal accumulator (Casini and Vidal, 2011), with particular relevance for the pre-action time (Macar et al., 1999; Iwasaki et al., 2019). In humans, the inter-individual variability in temporal estimation correlates with GABA concentration (Terhune et al., 2014) in the primary visual cortex. Interestingly, physical activity increases dopamine release in several brain areas (Dishman, 1997; Waters et al., 2005; Ma, 2008) and affects visual cortical activity and plasticity by modulating intra-cortical GABAergic inhibition (Sansevero et al., 2020). Moreover, the SMA is implicated in preparing sequential movements (Ohbayashi, 2021) and in gait control (Shibasaki et al., 2004). These common neural substrates suggest that physical exercise might affect time perception. A few studies reported that physical exercise could affect temporal judgments, inducing an overestimation of time intervals (Lambourne, 2012; Duncan et al., 2016; Behm and Carter, 2020; Petrizzo et al., 2022). For example, Petrizzo et al. (2022) performed a temporal and a numerosity discrimination task in which participants, while running, had to estimate whether the presented stimulus was the same or different from a memorized reference (600 ms interval or 24 dots). They found that distortion due to physical activity was specific to time but not numerosity and did not persist after the end of exercise. Differently, Lambourne (2012) compared the performance of healthy college students in a temporal generalization task and an episodic temporal generalization task before and during moderate physical exercise. Again, the author found a distortion of temporal perception, but no significant differences were detected on the episodic timing task.

However, these studies cannot disentangle the effect of exercise from other concurrent factors, such as heart rate modulation (Hawkes et al., 1962; Jamin et al., 2004; Schwarz et al., 2013), arousal (Dormal et al., 2018), and movement rhythmicity, which can influence the internal clock rate.

**Figure 1:**
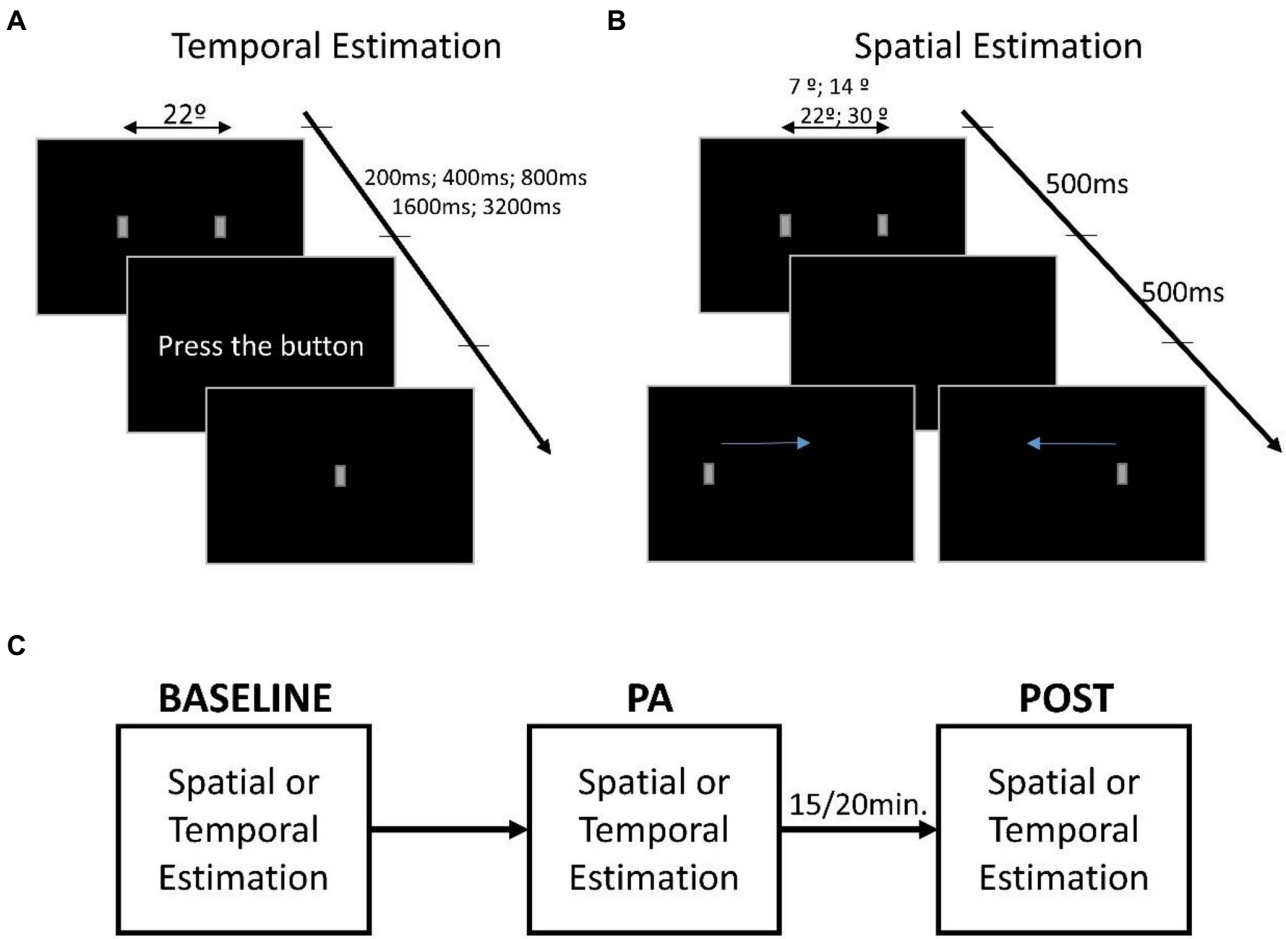
Representation of the estimation tasks. **(A)** Description of the time estimation task in which the first screen shows two small gray rectangles equidistant from the center of the screen. The presentation duration of stimuli can be 200, 400, 800, 1,600, and 3,200 ms, while the distance between rectangles is fixed. Once the stimuli have disappeared, the words “press the button” appear, only at which point the participant must press the space bar to reproduce the duration of the stimuli, making a small rectangle appear as visual feedback. **(B)** Description of the distance estimation task in which the first screen shows two small gray rectangles appearing at different distances (~7°, ~14°, ~22°, and ~30°), while the temporal presentation was fixed at 500 ms. Then only one of the two rectangles appears on the screen, and the participant’s task is to move the rectangle, using the arrows on the keyboard, to retrace the distance that separated the two rectangles. **(C)** The procedure used during the testing. The first session is the baseline used to evaluate estimation performance. Following is the physical activity (PA) session that started after the increase in heart rate. After ~15/20 min at the end of the PA session, the task was executed a third time for the POST session.

Here we investigate the impact of moderate physical exercise on a temporal estimation task in a group of adult participants by asking them to do the task before, during and after physical activity, Figure 1. Moreover, participants also performed a distance estimation task following the same experimental paradigm to control for possible perceptual distortions due to arousal and fatigue. Spatial representation can be modulated by arousal (Witt and Proffitt, 2005; Witt et al., 2008; Shiban et al., 2016), emotions (Stefanucci and Storbeck, 2009; Storbeck and Stefanucci, 2014; Cañal-Bruland et al., 2015), or action intentionality (Cutting and Vishton, 1995; Bhalla and Proffitt, 1999) but seems to be unaffected by GABAergic and dopaminergic modulation (Leonte et al., 2018). Therefore, we hypothesize that a genuine effect of physical exercise would be specific to time and not space perception, while arousal would have a generic impact on both perceptual domains.

## Materials and methods

### Participants

We recruited 16 participants (seven males, with an average age of 27.62, SD = 2.55). All participants had normal or corrected to normal vision and gave written informed consent before starting the experiment. The study was approved by the local health service ethics committee (Comitato Etico, ASL 3, Genova) and followed the tenets of the Declaration of Helsinki.

All participants, except one who lived a sedentary life, said they engaged in recreational physical activity (such as volleyball, football, or cross-fit). In the 6 months before the study, participants reported performing at least 2–3 h of sports per week. To estimate the sample size, we took as reference the results obtained by Dormal et al. (2018) in the ANOVA ran in the interaction between factors duration and block (*η*^2^ = 0.491). Using software G*power (Faul et al., 2007), we calculated the sample size we would need to use a two-way ANOVA, considering a significance level of *α* = 0.05 and power of (1−*β*) = 0.95. We found that a sample size of 8 participants would be needed.

### Stimuli

Visual stimuli consisted of a pair of gray rectangles with the longest side upright (dimension: ~3° × ~1.5°). Each rectangle was generated in MATLAB, using psychophysics toolbox extensions. Stimuli were displayed on ASUS VG248QE Monitor (24-inch) with a resolution of 1920 × 1080 and a refresh rate of 100 Hz. For the distance estimation, the stimuli always last 500 ms, while the distance between the rectangles could have been ~7°, ~14°, ~22°, and ~30°. For the temporal estimation, the distance between the rectangles was ~22°, while the presentation duration could vary between 200, 400, 800, 1,600, and 3,200 ms. See Figure 1 for a diagram of the experimental protocol.

The H7 heart rate sensor by Polar was used to record the heart rate. The sensor was applied at the diaphragm level using the appropriate band and connected *via* Bluetooth to the app Polar Beat, through which the experimenter could monitor the heart rate. Physical activity was performed stationary cycling, using a rehabilitation pedalboard (model Welly M, EVERFIT).

### Procedure

The experiment consisted of two sessions: one performed in the morning and one in the afternoon of the same day. Each participant performed the temporal estimation task in one session and the distance estimation task in the other. The session order was randomized among participants. Before each session, participants wore the heartbeat sensor, sat in front of the monitor, and with the experimenter’s help, positioned the pedalboard at the proper distance to the pedal. Afterward, the experimenter adjusted the monitor’s position at ~70 cm from the participant’s head.

### Distance estimation task

Two rectangles were presented at one of the four possible distances for 500 ms. Once the rectangles disappeared, a new rectangle appeared randomly on the left or right of the screen. The participant was asked to reproduce the distance that separated the previous rectangles by moving the one on the screen using the left and right arrows on the keyboard, Figure 1B. Once positioned in the rectangle, the participant pressed the enter button to confirm the response and start the subsequent trial.

### Temporal estimation task

The two rectangles always appeared on the screen at the same distance (~22°) but for a randomized duration. Once the rectangles disappeared, the participant had to press the space bar on the keyboard, reproducing the previous presentation duration of the rectangles, Figure 1A. Visual feedback was provided by a rectangle presented in the center of the screen while participants pressed the space bar key.

Both tasks were repeated three times (sessions). In the first session (baseline), participants performed the task while sitting comfortably on the chair. In the second session (physical activity, PA), participants performed the task while cycling in the second session. The experimenter monitored the heart rate to be maintained at around 40 beats per minute (bpm) above the basal rate (measured for each participant at rest) to ensure that the level of exercise was kept within a light to moderate level (Strath et al., 2013), and that the cycling effort was adequate and constant during the test. Because of our participants’ age and baseline heart rate, +40 bpm allowed all participants to achieve a light to moderate level of physical exercise (bpm range during physical activity: 40–60% of the maximum heart rate (Tanaka et al., 2001)). Finally, the third session (POST) was performed at rest (i.e., no cycling) around 15 min after the end of the PA session, when the heart rate had returned to the basal rate.

Each duration/distance was repeated 18 times for 90 trials for the temporal estimation and 72 trials for the distance estimation for each session. All experiments lasted around 1 h and a half.

Throughout the experiment, the average heart rate for each session was recorded. Moreover, at the end of the experiment, each participant was asked about the frequency of physical exercise per week for the last 3 months.

### Data analysis

For each task and session, we calculated the average bias in estimating the duration/distance of the visual stimuli by subtracting the perceived duration/distance from the physical duration/distance. We ran a Lilliefors (Kolmogorov–Smirnov—using rStudio Packages “normtest”) test to check the normality of the sample for each condition in each task. All tests respect the assumption of normality.

Moreover, we defined the Weber Fraction (WF) as perceptual precision, calculated as the ratio of the standard deviation to the perceived duration/distance.

Using the bias and successively the WF as a dependent variable, we run two separate two-way repeated measure ANOVAs, for each variable, for the distance and temporal estimation. For the distance estimation, we had as between factors Session (baseline, PA and POST) and distance (7°, 14°, 22°, and 30°), while for the temporal estimation, we used as between factor Session (baseline, PA and POST) and Duration (200 ms, 400 ms, 800 ms, 1,600 ms, and 3,200 ms).

In a second step, to investigate the effects of physical activity in more detail, we computed the under- or overestimation as proportional shifts, defined as the difference between the perceived duration (or Distance) and the real physical value, normalized by the real value.


$$\mathrm{Bias}\%=\left(\frac{\mathrm{perceived value}-\mathrm{real value}}{\mathrm{real value}}\right)\times 100$$

Using this value as a dependent variable, we run two separate two-way repeated measure ANOVAs for the distance and temporal estimation. For the distance estimation, we had as between factors Session (baseline, PA and POST) and Distance (7°, 14°, 22°, and 30°), while for the temporal estimation, we used as between factor Session (baseline, PA and POST) and Duration (200, 400, 800, 1,600, and 3,200 ms).

Then we calculated the percentage of change of the bias relative to the baseline by subtracting the percentage of bias observed in the PA and POST sessions from that observed in the baseline session for each participant. A delta equal to zero indicates no effect from physical activity, so each delta was compared with zero, using a one-sample t-test against zero.

Using the bias percentage, we directly compared the performance of distance and temporal estimation tasks in a third analysis. We averaged the percentage across all the durations/distances to do so. We used this value as a dependent variable in a two-way repeated measure ANOVA with between factors Session (baseline, PA and POST) and Task (spatial and temporal).

As a final analysis, we correlated the average heart rate of each participant with the average bias obtained in the time estimation task for each of the three sessions using a Person correlation analysis.

Analysis of the HR in each condition is reported in the Supplementary materials.

All *p*-values for the t-test analysis were corrected for multiple comparisons (Holms), and Cohen’s *d* gives the effect size. All ANOVAs and t-tests were done using JASP software (Love et al., 2019).

## Results

**Figure 2:**
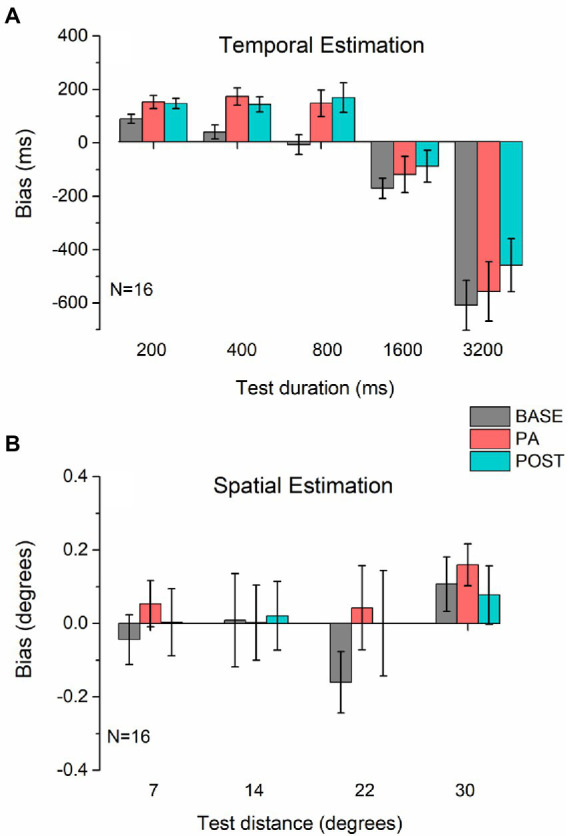
Estimation bias. Average bias in temporal **(A)** and spatial **(B)** estimation measured before (BASE, gray bars), during (PA, red bars), and after (POST, blue bars) physical activity. In both panels, values above the dotted line (0) indicate an overestimation of time **(A)** or distance **(B)**, while values below zero indicate underestimation. Error bars represent 1 ± SEM.

We found that physical activity clearly affected the temporal estimation task (Figure 2A), an effect that persisted even in the POST session. For all durations, physical activity induced an overestimation of the real duration. A repeated measures ANOVA revealed a significant main effect for Duration (*F*_4,60_ = 38.85, *p* < 0.001, *η*^2^ = 0.72) and Session (*F*_2,30_ = 6.32, *p* < 0.01, *η*^2^ = 0.3), but not a significant interaction between the two (*F*_8,120_ = 1.2, *p* = 0.31, *η*^2^ = 0.07), because the effect of physical activity was constant for all durations tested. *Post hoc* tests pooling all pedestal durations showed a significant difference between baseline and PA (*t* = 2.54, *p* < 0.05, *d* = 0.64), and baseline and PO (*t* = 3.72, *p* < 0.01, *d* = 0.93), but not between PA and PO (*t* = 0.66, *p* = 0.52, *d* = 0.16), confirming that there is an effect of physical activity and that this effect persists afterward. The persistence of the effect in the POST session rules out a possible impact of the rhythmicity of the cycling movement on the temporal distortion observed during physical exercise. Instead, for the distance estimation task, on average, participants were extremely accurate across all sessions (Figure 2B). The physical activity produced a slight, non-significant, bias leading to an overestimation of distance in almost all conditions, yet this effect was not maintained in the POST session. Consistently, the rm-ANOVA on the distance estimation was not significant for either of the main factors (Session: *F*_2,30_ = 1.023, *p* = 0.37, *η*^2^ = 0.012; Distance: *F*_3,45_ = 1.13, *p* = 0.35, *η*^2^ = 0.03) or the interaction (*F*_6,90_ = 0.52, *p* = 0.79, *η*^2^ = 0.013).

Regarding the percentage of Bias in the temporal task, similar to the previous analysis we find both the main effects significant (Duration: *F*_4, 60_ = 49.02, *p* < 0.001, *η*^2^ = 0.77; Session: *F*_2, 30_ = 8.79, *p* < 0.001, *η*^2^ = 0.37), as well as the interaction between Session and Duration (*F*_8, 120_ = 3.59, *p* < 0.001, *η*^2^ = 0.19). While for the spatial task the results appear to be unchanged, as none of the main effects (Distance: *F*_3, 45_ = 0.22, *p* = 0.88, *η*^2^ = 0.014; Session: *F*_2, 30_ = 1.04, *p* = 0.37, *η*^2^ = 0.06) or interaction (*F*_8, 120_ = 0.43, *p* = 0.86, *η*^2^ = 0.03) are significant.

**Figure 3:**
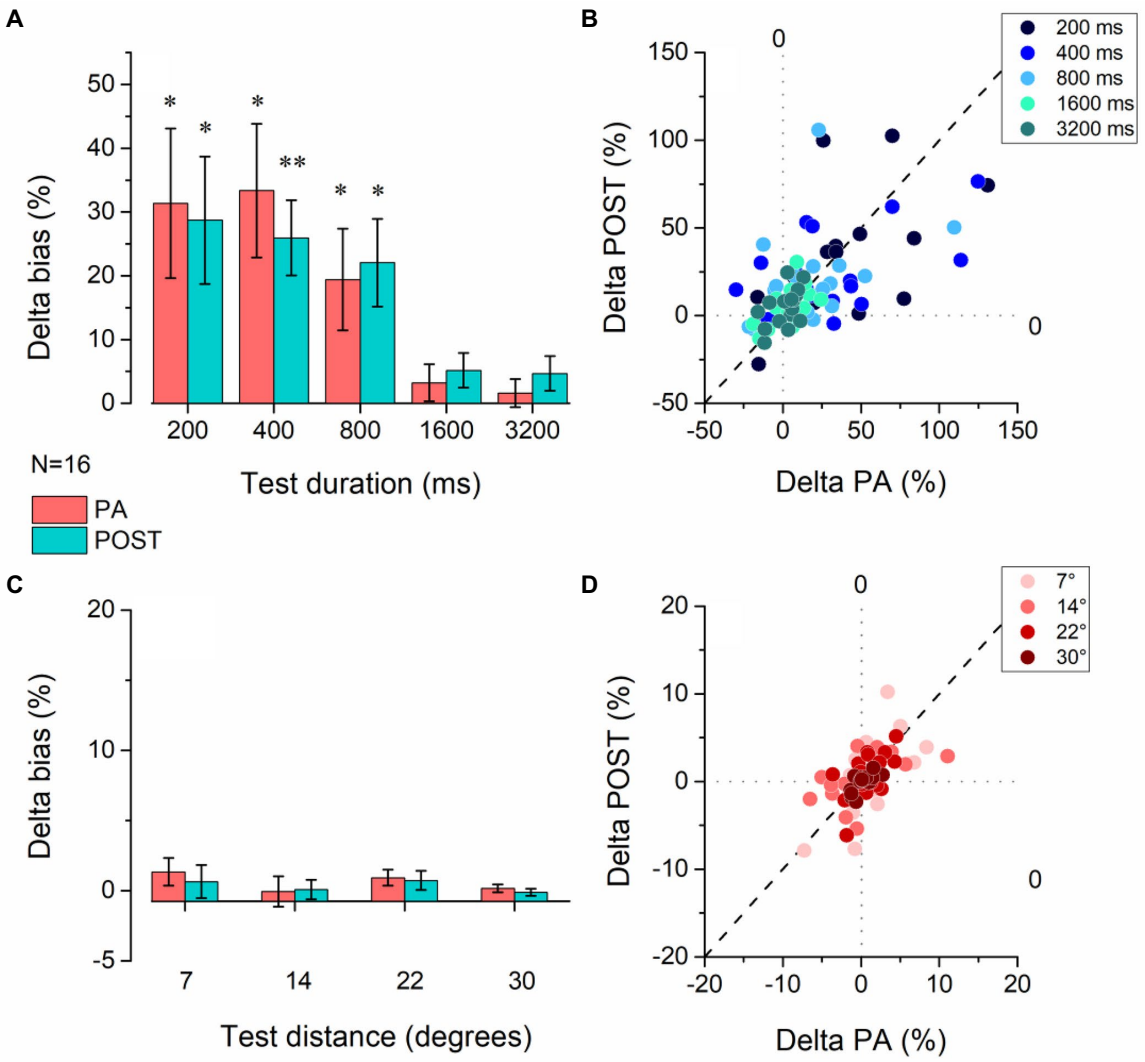
Effect of physical activity on temporal and spatial estimation. **(A)** Average delta values for the temporal task were obtained by subtracting the bias observed in the baseline session before physical activity from the experimental sessions measured during (PA – red bars) and after (POST –red) physical activity. The delta was calculated for each duration of the time estimation task. Error bars represent SEM. **(B)** Scatter plot of the delta values obtained for each participant during physical activity (delta PA) vs. after physical activity (delta POST) for all interval pedestals tested. **(C)** Average delta values for the spatial task were obtained by subtracting the bias observed in the baseline session before physical activity from the experimental sessions measured during (PA – red bars) and after (POST –red) physical activity. The delta was calculated for each duration of the time estimation task. Error bars represent SEM. **(D)** Scatter plot of the delta values obtained for each participant during physical activity (delta PA) vs. after physical activity (delta POST) for all distances tested. *p < 0.05, **p < =0.01 (FDR correction for multiple comparisons applied to the *p*-values).

We further characterize the effect of physical activity on temporal estimation by analyzing the % Deltas from the baseline for PA and POST sessions across pedestal durations (Figures 3A,B). The physical activity effect was significantly different from zero in the PA and POST sessions for the durations below the second but not for durations above the second (Figure 3A). Table 1 summarizes all the statistical results for the delta. For the three pedestal intervals tested in the sub-second range (200, 400, and 800 ms, Figure 3B), physical exercise induced a substantial overestimation of time (200 ms pedestal delta = 31.36.7 ± 47.04%, 400 ms pedestal delta = 33.35 ± 41.99%, 800 ms pedestal delta = 19.39 ± 31.89%), which was maintained in the POST session (after exercise) once the heart rate returned to baseline levels. No significant difference was found for the spatial estimation task (Figures 3C,D). A summary of the results is in Table 1.

**Table 1:** Summary of *t*-tests against zero of % Deltas for both the temporal and spatial estimation task.

Condition	Value ms/° (SD)	*t*	df	*p*	Cohen’s *d*
Delta PA 200 ms	63 (94)	2.53	15	0.036	0.67
Delta PA 400 ms	133 (168)	2.83	15	0.02	0.79
Delta PA 800 ms	155 (255)	2.25	15	0.046	0.61
Delta PA 1600 ms	51 (185)	1.03	15	0.31	0.28
Delta PA 3200 ms	51 (285)	0.79	15	0.48	0.18
Delta POST 200 ms	57 (80)	2.55	15	0.03	0.72
Delta POST 400 ms	104 (94)	4.11	15	0.01	1.1
Delta POST 800 ms	176 (220)	3.83	15	0.02	0.8
Delta POST 1600 ms	83 (174)	1.87	15	0.1	0.47
Delta POST 3200 ms	149 (348)	1.83	15	0.13	0.43
Delta PA 7°	0.098 (0.28)	1.37	15	0.76	0.34
Delta PA 14°	−0.006 (0.63)	0.04	15	0.97	0.01
Delta PA 22°	0.2 (0.49)	1.66	15	0.76	0.41
Delta PA 30°	0.05 (0.32)	0.65	15	0.92	0.16
Delta POST 7°	0.047 (0.34)	0.55	15	0.92	0.14
Delta POST 14°	0.012 (0.39)	0.12	15	0.97	0.03
Delta POST 22°	0.16 (0.59)	1.09	15	0.77	0.27
Delta POST 30°	−0.03 (0.29)	0.41	15	0.92	0.1

All *p* values are corrected for multiple comparisons (FDR).

Moreover, we directly compared the percentage of Bias between the temporal and the spatial task. Both main effects were significant (Task: *F*_1, 15_ = 29.81, *p* < 0.001, *η*^2^ = 0.66; Session: *F*_2, 30_ = 9.28, *p* < 0.001, *η*^2^ = 0.38), and the interaction between Session and Task (*F*_2, 30_ = 8.19, *p* < 0.01, *η*^2^ = 0.35). *Post hoc* analysis revealed no significant difference between the two tasks in the PRE sessions (*t* = 1.25, *p* = 1, CI [−18.16 7.77]), meaning that at the beginning of the experiment the percentage of Bias was comparable. Nevertheless, we found a significant difference between the tasks in the PA session (*t* = 5.41, *p* < 0.001, CI [−35.34–9.41]), and in the POST session (*t* = 5.36, *p* < 0.001, CI [−35.13–9.2]). In the temporal task, the percentage of Bias in the PRE session was significantly different from the percentage in the PA (*t* = 5.18, *p* < 0.001, CI [−28.28–7.29]) and POST session (*t* = 5.04, *p* < 0.001, CI [−27.81–6.82]), while no difference was found in the spatial task neither between the PRE and PA sessions (*t* = 0.18, *p* = 1, CI [−11.09 9.89]) nor between PRE and POST (*t* = 0.1, *p* = 1, CI [−10.83 10.15]).

**Figure 4:**
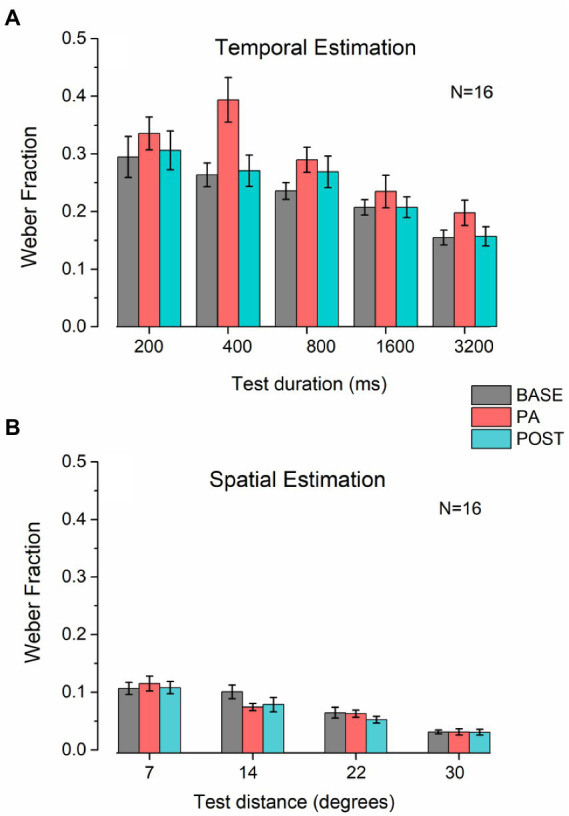
Weber Fraction on temporal and spatial estimation. **(A)** Bar plots represent the average Weber fraction for each session and duration tested in the temporal estimation task. Error bars represent SEM. **(B)** Bar plots represent the average Weber fraction for each session and distance tested in the spatial estimation task. Error bars represent SEM.

To evaluate perceptual precision during the different conditions, we run ANOVAs using the Weber Fraction (WF) as the dependent variable in both tasks (spatial and temporal). For the temporal estimation task (Figure 4A), we found a significant main effect of Session (*F*_2, 30_ = 4.89, *p* < 0.05, *η*^2^ = 0.25) and Duration (*F*_4, 60_ = 12.56, *p* < 0.01, *η*^2^ = 0.46), but only a trend for the interaction between the factors (*F*_8, 120_ = 1.96, *p* = 0.06, *η*^2^ = 0.12). *Post hoc* analysis on the main factors revealed that there is a significant difference between the PRE and PA condition (*t* = 3.13, *p* < 0.05, *d* = 0.78), but no difference between PRE and POST (*t* = 1.6, *p* = 0.24, *d* = 0.4), nor between POST and PA (*t* = 1.52, *p* = 0.24, *d* = 0.38), while the durations in the sub-second range were significantly different between the one over the second, but not different between each other (see Table 2 for a summary of results).

**Table 2:** Summary of *t*-tests for the WFs in the temporal estimation task.

Condition	*t*	*p*	Cohen’s d
200 ms vs. 400 ms	0.11	1	0.03
200 ms vs. 800 ms	0.58	1	0.14
200 ms vs. 1,600 ms	3.72	<0.01	0.93
200 ms vs. 3,200 ms	5.54	<0.001	1.39
400 ms vs. 800 ms	0.47	1	0.12
400 ms vs. 1,600 ms	3.61	<0.02	0.9
400 ms vs. 3,200 ms	5.44	<0.001	1.36
800 ms vs. 1,600 ms	3.14	<0.05	0.79
800 ms vs. 3,200 ms	4.96	<0.001	1.24
1,600 ms vs. 3,200 ms	1.82	0.39	0.45

All *p* values are corrected for multiple comparisons.

For the spatial estimation task (Figure 4B), the only significant factor was Distance (*F*_3, 45_ = 28.43, *p* < 0.001, *η*^2^ = 0.65), and not for Session (*F*_2, 305_ = 1.44, *p* = 0.25, *η*^2^ = 0.088) nor the interaction (*F*_6, 90_ = 1.84, *p* = 0.1, *η*^2^ = 0.11).

**Figure 5:**
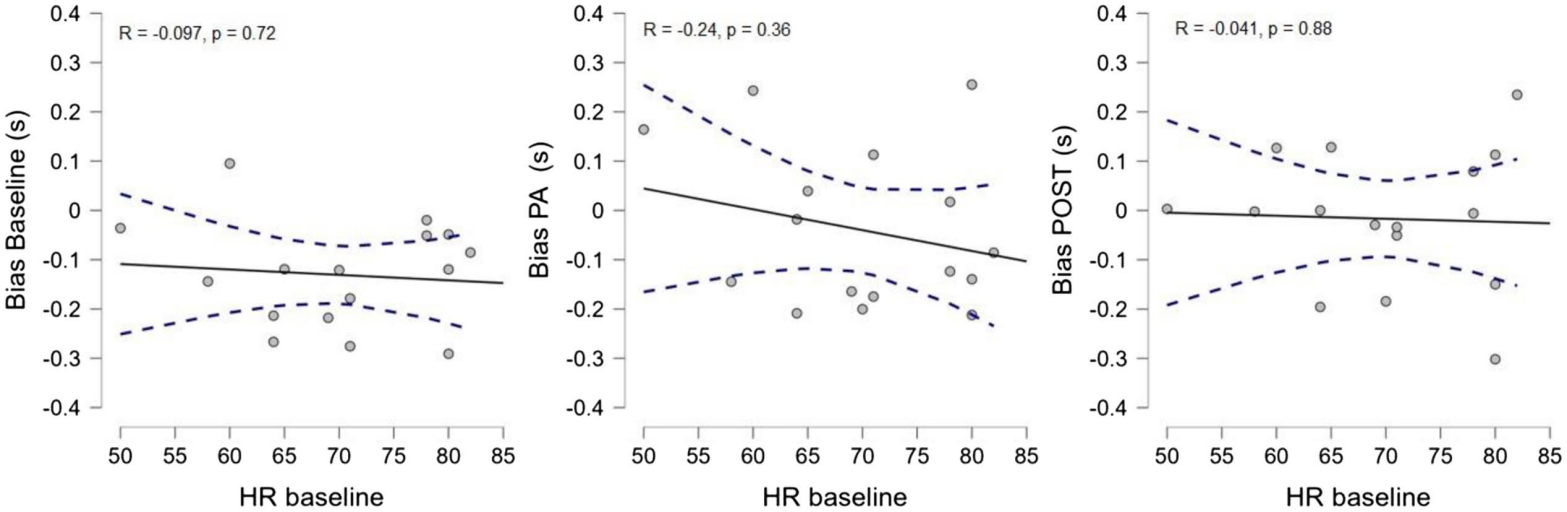
Correlation between the average HR in the baseline condition and the average temporal bias for each experimental session.

Finally, to further check for a possible contribution of the increase in heart rate induced by physical exercise to the observed effect, we correlated the heart rate in the baseline condition with the average bias of the time estimation task in each experimental session (Figure 5). We found no correlation between baseline heart rate and temporal estimation bias in either condition (baseline: *R* = −0.097, *p* = 0.721, CI 95% [−0.565 0.419]; PA: *R* = −0.24, *p* = 0.36, CI 95% [−0.66 0.285]; POST: *R* = −0.041, *p* = 0.88, CI 95% [−0.526 0.46]), indicating that the temporal distortion observed during and after physical activity was not due to the heart rate acceleration induced by exercise.

## Discussion

We investigated the influence of physical activity on time perception by having participants perform temporal estimation tasks before, during, and after moderate physical exercise. We showed that moderate physical activity substantially affected temporal estimation, inducing a robust overestimation of durations under 1 s. Notably, the effect persisted even after the end of physical activity when heart rate returned to baseline levels. There was no correlation between baseline heart rate and temporal bias, indicating that the effect of physical exercise was not due to the change in heart rate. The persistence of the effect after the end of motor activity also rules out the possibility that the rhythmicity of pedaling and its variation in speed during physical activity might influence temporal estimation (Gupta et al., 2020). Moreover, since Weber Fractions measured in the POST condition were indistinguishable from those obtained in the baseline condition, the persistence of the effect after the end of exercise also rules out the possibility that the temporal distortion induced by exercise might be related to lower precision in the estimation task during physical activity. Finally, the perceptual distortion induced by exercise was specific to temporal estimation; as in the control experiment, distance estimation was unaffected by physical activity.

The temporal distortion induced by physical activity is in line with previous reports (Lambourne, 2012; Schwarz et al., 2013; Dormal et al., 2018; Behm and Carter, 2020; Petrizzo et al., 2022). However, in the study by Dormal et al. (2018), the authors conclude that the temporal distortion is not strictly related to physical activity *per se* but linked to other uncontrolled attentional or mnestic factors. This result is confirmed by other studies showing how perception (Mather et al., 2016), and even temporal perception (Gable et al., 2016), can be altered by the participant’s emotional state. Nevertheless, we believe that our results provide evidence of a genuine effect of physical activity while performing the task, which cannot be attributed to arousal, fatigue, or emotional state as these factors would also affect spatial estimation (Stefanucci and Storbeck, 2009; Geuss et al., 2010; Storbeck and Stefanucci, 2014; Cañal-Bruland et al., 2015; Shiban et al., 2016).

In their experiment, Petrizzo et al. (2022) found a consistent (about 20%) overestimation of time (600 ms interval), but not numerosity, during aerobic physical activity (running on a treadmill). The bias induced by exercise disappeared soon after the end of physical activity, when heart rate had not yet returned to baseline levels, ruling out a significant contribution of heart rate and arousal increase to the observed effect. From this result, the authors conclude that temporal distortion is induced by physical activity, particularly by the rhythmic movement during running (Petrizzo et al., 2022). Our study extends Petrizzo et al. (2022) results, investigating the effect of physical activity on time estimation over a wide range of time intervals. Moreover, unlike this previous report, we found that the temporal distortion persisted for at least 15–20 min after the end of physical activity when heart rate had returned to baseline levels. This result differs from Petrizzo et al. (2022) and indicates that movement rhythmicity is not the sole contributor to the observed effect. There are two major differences between our study and Petrizzo et al.’s: the type of physical exercise (cycling vs. running) and the level of exercise (moderate vs. vigorous). In our paradigm, physical activity (and, therefore, movement) was confined to the lower limbs, and the head position was stable during the estimation task, as participants were sitting on a chair while pedaling. In contrast, running on a treadmill involves movement of the whole body, as well as rhythmic head movements, which call for automatic rhythmic eye movements to guarantee visual stability and might contribute to perceptual distortions. Moreover, in our study, physical exercise was moderate (about 50% of the maximum heart rate), while in Petrizzo et al. (2022), physical exercise was vigorous (80% of the maximum heart rate). Moderate and vigorous levels of physical exercise have different effects on perception and cognitive functions (Cantelon and Giles, 2021). For example, while moderate physical exercise improves response times for executive functions tasks, vigorous exercise impairs accuracy in the same tasks (Cantelon and Giles, 2021). Similarly, Marchant et al. (2020) reported that moderate, but not vigorous physical exercise improved short-term memory recall without increasing false recall. This evidence indicates different effects of exercise intensity on cognitive functions, with more consistent beneficial effects being observed for moderate levels of exercise (Cantelon and Giles, 2021). These two differences between our two paradigms might explain the different results obtained after the end of physical activity.

Another major point of our results is that while temporal overestimation was found for all durations tested, the effect was significant only for durations below one second. Mauk and Buonomano (2004) hypothesized the existence of four different temporal scales devoted to the processing of temporal information: microseconds (Covey and Casseday, 1999), milliseconds (Buonomano and Buonomano and Karmarkar, 2002), seconds (Gibbon, 1977), and circadian rhythms. Millisecond processing would be fundamental for speech processing, motion detection, and motor coordination. Instead, processing in the seconds’ range refers to the conscious perception of time. Since movement involves prolonged physical change over time, motor control and timing are related closely in the order of milliseconds. This link could be the basis of our finding: physical activity only affects the millisecond range. Continuous motor activation during cycling, and thus muscle exertion, could increase internal noise causing an overestimation for shorter temporal durations, but leave the temporal estimate at the conscious level almost intact. This hypothesis might be supported by the fact that, across all conditions, we observed higher Weber Fractions (and therefore lower precision) for durations in the millisecond range. However, the lack of correlation that we found between Weber fractions and the percentage of bias in temporal estimates across all pedestal durations in the POST condition (Supplementary Figure 3) indicates that perceptual precision and the temporal distortion induced by physical exercise are not related.

According to the classical models of temporal perception (Treisman, 1963; Gibbon, 1977; Zakay and Block, 1996), another possibility is that physical activity could directly influence an internal pacemaker by increasing the rate of accumulated pulses and leading to an overestimation of temporal intervals. However, we did not find a correlation between temporal bias and heart rate, which can be considered a potential physiological correlate of the pacemaker (Duncan et al., 2016; van der Ham et al., 2019; Behm and Carter, 2020). Moreover, it is essential to highlight that a change in the internal pacemaker would have an augmentative property, i.e., time distortion would increase with the stimulus duration (Zakay and Block, 1997; Gil and Droit-Volet, 2012). That physical activity induces a temporal distortion only for durations below one-second further points to a genuine effect of physical exercise on temporal estimation and no interference between the heart rate and the internal clock.

What alternative theory could be if the classical models cannot fully explain the observed effect? The striatal beat-frequency model (SBF) proposes that temporal perception lies in the timing of various complex behaviors and provides a network of neural regions involved in it (Merchant et al., 2013). This model proposes that the clock speed is modulated by activity levels of dopamine-glutamate in the substantia nigra compacta and ventral-tegmental area-cortical pathways (Matell and Meck, 2004; Meck, 2005). In this theory, time distortion is ascribed to context-dependent activation dynamics that interfere with neural network activity. This might explain our results on temporal distortion during the physical activity session (PA) and the persistence of the effect during the POST session. There is extensive literature on the relationship between physical activity and the dopaminergic system (Foley and Fleshner, 2008; Knab and Lightfoot, 2010). Physical exercise increases the amount of dopamine released and metabolized in some brain areas (Dishman, 1997; Waters et al., 2005; Ma, 2008), underlying the improvement observed, for example, in neuronal plasticity (Foley and Fleshner, 2008) and cognitive function (Singh-Manoux et al., 2005; Voss et al., 2011). Therefore, a possible explanation of the temporal bias during our experiment is that the cycling induced an increase in dopamine production that interferes with the network of brain areas proposed by the SBF model, causing a speed-up of the internal clock leading to the temporal bias. Another point in favor of this idea is the persistence of the effect in the POST session, i.e., the dopamine release produced by physical activity persists despite the newfound homeostasis of the body system by continuing to alter the internal clock.

Finally, another neural mechanism possibly involved in the reported effect could be a modulation of GABAergic inhibition. Evidence from animal models shows that physical exercise increases visual cortical activity (Niell and Stryker, 2010) and enhances visual plasticity (Kaneko and Stryker, 2014) by reducing intra-cortical GABA concentration (Baroncelli et al., 2012; Sansevero et al., 2020) through a specific dis-inhibitory circuit in the primary visual cortex (Stryker, 2014; Fu et al., 2015). Interestingly, the concentration of GABA in the primary visual cortex of adult humans correlates with the perceived duration of time intervals in the sub-second range (Terhune et al., 2014) - the underestimation of temporal intervals is associated with higher GABA concentrations, and the administration of synthetic GABA alters temporal visual attention while not affecting spatial–visual attention (Leonte et al., 2018). Therefore, our finding that physical activity induces an overestimation of time intervals shorter than one second while not affecting distance estimation is consistent with reducing intra-cortical GABAergic inhibition induced by physical exercise.

## Conclusion

In the present study, we have shown (1) that the influence of physical activity is specific to the temporal domain, causing a general overestimation of the tested durations, and the heart rate does not correlate with it. (2) The effect on the temporal task can be related to the increased production of the dopaminergic system, which causes an increase in the speed of the internal clock or reduces intra-cortical GABAergic inhibition. (3) Physical activity influenced only temporal perception in milliseconds probability because of motor control.

## Data availability statement

The original contributions presented in the study are publicly available. This data can be found here: https://zenodo.org/record/7035801.

## Ethics statement

The studies involving human participants were reviewed and approved by Comitato Etico, ASL 3, Genova. The patients/participants provided their written informed consent to participate in this study.

## Author contributions

AT, CL, and MG conceived and designed the experiment. AT performed the data collection and data analysis. All authors contributed to the article and approved the submitted version.

## Funding

This project has received funding from the European Research Council (ERC) under the European Union’s Horizon 2020 research and innovation program (grant agreement No 948349 – MYSpace, and No 948366 – HOPLA) and the French National Research Agency (ANR: AAPG 2019 JCJC, grant agreement ANR-19-CE28-0008, PlaStiC, and FrontCog grant ANR-17-EURE-0017).

## Conflict of interest

The authors declare that the research was conducted in the absence of any commercial or financial relationships that could be construed as a potential conflict of interest.

## Publisher’s note

All claims expressed in this article are solely those of the authors and do not necessarily represent those of their affiliated organizations, or those of the publisher, the editors and the reviewers. Any product that may be evaluated in this article, or claim that may be made by its manufacturer, is not guaranteed or endorsed by the publisher.

## References

[ref1] Ayaz A. Saleem A. B. Schölvinck M. L. Carandini M. (2013). Locomotion controls spatial integration in mouse visual cortex. Curr. Biol. 23, 890–894. doi: 10.1016/j.cub.2013.04.012, PMID: 23664971 PMC3661981

[ref2] Baek S.-S. (2016). Role of exercise on the brain. J. Exerc. Rehabil 12, 380–385. doi: 10.12965/jer.1632808.404, PMID: 27807514 PMC5091051

[ref3] Baroncelli L. Bonaccorsi J. Milanese M. Bonifacino T. Giribaldi F. Manno I.. (2012). Enriched experience and recovery from amblyopia in adult rats: impact of motor, social and sensory components. Neuropharmacology 62, 2388–2397. doi: 10.1016/j.neuropharm.2012.02.010, PMID: 22532989

[ref4] Behm D. G. Carter T. B. (2020). Effect of exercise-related factors on the perception of time. Front. Physiol. 11:770. doi: 10.3389/fphys.2020.00770, PMID: 32733275 PMC7357302

[ref5] Bhalla M. Proffitt D. R. (1999). Visual–motor recalibration in geographical slant perception. J. Exp. Psychol. Hum. Percept. Perform. 25, 1076–1096. doi: 10.1037/0096-1523.25.4.1076, PMID: 10464946

[ref100] Brisswalter J. Collardeau M. René A. (2002). Effects of acute physical exercise characteristics on cognitive performance. Sport. Med. 32, 555–566. doi: 10.2165/00007256-200232090-00002 12096929

[ref6] Bullock T. Elliott J. C. Serences J. T. Giesbrecht B. (2017). Acute exercise modulates feature-selective responses in human cortex. J. Cogn. Neurosci. 29, 605–618. doi: 10.1162/jocn_a_01082, PMID: 27897672

[ref7] Buonomano D. V. Karmarkar U. R. (2002). Book review: how do we tell time? Neuroscientist 8, 42–51. doi: 10.1177/107385840200800109 11843098

[ref8] Cañal-Bruland R. Aertssen A. M. Ham L. Stins J. (2015). Size estimates of action-relevant space remain invariant in the face of systematic changes to postural stability and arousal. Conscious. Cogn. 34, 98–103. doi: 10.1016/j.concog.2015.04.006, PMID: 25913547

[ref9] Cantelon J. A. Giles G. E. (2021). A review of cognitive changes during acute aerobic exercise. Front. Psychol. 12:653158. doi: 10.3389/fpsyg.2021.653158, PMID: 34975602 PMC8716584

[ref10] Cao L. Händel B. (2019). Walking enhances peripheral visual processing in humans. PLoS Biol. 17:e3000511. doi: 10.1371/journal.pbio.3000511, PMID: 31603894 PMC6808500

[ref11] Casini L. Vidal F. (2011). The SMAs: neural substrate of the temporal accumulator? Front. Integr. Neurosci. 5:35. doi: 10.3389/fnint.2011.00035, PMID: 21886611 PMC3154296

[ref12] Collignon O. Champoux F. Voss P. Lepore F. (2011). Sensory rehabilitation in the plastic brain. Prog. Brain Res. 191, 211–231. doi: 10.1016/B978-0-444-53752-2.00003-5 21741554

[ref13] Coull J. T. Cheng R.-K. Meck W. H. (2011). Neuroanatomical and neurochemical substrates of timing. Neuropsychopharmacology 36, 3–25. doi: 10.1038/npp.2010.113, PMID: 20668434 PMC3055517

[ref14] Covey E. Casseday J. H. (1999). Timing in the auditory system of the bat. Annu. Rev. Physiol. 61, 457–476. doi: 10.1146/annurev.physiol.61.1.457, PMID: 10099697

[ref15] Cutting J. E. Vishton P. M. (1995). “Perceiving layout and knowing distances: the integration, relative potency, and contextual use of different information about depth,” in Perception of Space and Motion. eds. Epstein W. Rogers S. (San Diego, CA: Academic Press), 69–117.

[ref16] Dishman R. K. (1997). Brain monoamines, exercise, and behavioral stress: animal models. Med. Sci. Sport. Exerc. 29, 63–74. doi: 10.1097/00005768-199701000-00010 9000157

[ref17] Dormal V. Heeren A. Pesenti M. Maurage P. (2018). Time perception is not for the faint-hearted? Physiological arousal does not influence duration categorisation. Cogn. Process. 19, 399–409. doi: 10.1007/s10339-017-0852-3 29260437

[ref18] Droit-Volet S. Gil S. (2009). The time–emotion paradox. Philos. Trans. R. Soc. B Biol. Sci. 364, 1943–1953. doi: 10.1098/rstb.2009.0013, PMID: 19487196 PMC2685815

[ref19] Duncan M. J. Smith M. Bryant E. Eyre E. Cook K. Hankey J.. (2016). Effects of increasing and decreasing physiological arousal on anticipation timing performance during competition and practice. Eur. J. Sport Sci. 16, 27–35. doi: 10.1080/17461391.2014.979248, PMID: 25469534

[ref20] Faul F. Erdfelder E. Lang A.-G. Buchner A. (2007). G*Power 3: a flexible statistical power analysis program for the social, behavioral, and biomedical sciences. Behav. Res. Methods 39, 175–191. doi: 10.3758/BF03193146, PMID: 17695343

[ref21] Foley T. E. Fleshner M. (2008). Neuroplasticity of dopamine circuits after exercise: implications for central fatigue. NeuroMolecular Med. 10, 67–80. doi: 10.1007/s12017-008-8032-3, PMID: 18274707

[ref22] Fu Y. Kaneko M. Tang Y. Alvarez-Buylla A. Stryker M. P. (2015). A cortical disinhibitory circuit for enhancing adult plasticity. eLife 4:e05558. doi: 10.7554/eLife.05558, PMID: 25626167 PMC4337686

[ref200] Gable P. A. Neal L. B. Poole B. D. (2016). Sadness speeds and disgust drags: Influence of motivational direction on time perception in negative affect. Motiv. Sci. 2, 238–255. doi: 10.1037/mot0000044

[ref23] Geuss M. N. Stefanucci J. K. de Benedictis-Kessner J. Stevens N. R. (2010). A balancing act: physical balance, through arousal, influences size perception. Atten. Percept. Psychophys. 72, 1890–1902. doi: 10.3758/APP.72.7.1890, PMID: 20952786 PMC3298363

[ref24] Gibbon J. (1977). Scalar expectancy theory and Weber’s law in animal timing. Psychol. Rev. 84, 279–325. doi: 10.1037/0033-295X.84.3.279

[ref25] Gil S. Droit-Volet S. (2012). Emotional time distortions: the fundamental role of arousal. Cogn. Emot. 26, 847–862. doi: 10.1080/02699931.2011.625401, PMID: 22296278

[ref26] Gupta D. S. Banerjee A. Roy D. Piras F. (2020). Editorial: temporal structure of neural processes coupling sensory, motor and cognitive functions of the brain. Front. Comput. Neurosci. 14:73. doi: 10.3389/fncom.2020.00073, PMID: 33041775 PMC7522307

[ref27] Hawkes G. R. Joy R. J. T. Evans W. O. (1962). Autonomic effects on estimates of time: evidence for a physiological correlate of temporal experience. J. Psychol. 53, 183–191. doi: 10.1080/00223980.1962.9916563 13905502

[ref28] Iwasaki M. Noguchi Y. Kakigi R. (2019). Neural correlates of time distortion in a preaction period. Hum. Brain Mapp. 40, 804–817. doi: 10.1002/hbm.24413, PMID: 30276935 PMC6865754

[ref29] Jamin T. Joulia F. Fontanari P. Giacomoni M. Bonnon M. Vidal F.. (2004). Apnea-induced changes in time estimation and its relation to bradycardia. Aviat. Space Environ. Med. 75, 876–880. 15497368

[ref30] Kaneko M. Stryker M. P. (2014). Sensory experience during locomotion promotes recovery of function in adult visual cortex. eLife 3:e02798. doi: 10.7554/eLife.02798, PMID: 24970838 PMC4070284

[ref31] Knab A. M. Lightfoot J. T. (2010). Does the difference between physically active and couch potato lie in the dopamine system? Int. J. Biol. Sci. 6, 133–150. doi: 10.7150/ijbs.6.133 20224735 PMC2836544

[ref32] Lambourne K. (2012). The effects of acute exercise on temporal generalization. Q. J. Exp. Psychol. 65, 526–540. doi: 10.1080/17470218.2011.605959, PMID: 21936647

[ref33] Leonte A. Colzato L. S. Steenbergen L. Hommel B. Akyürek E. G. (2018). Supplementation of gamma-aminobutyric acid (GABA) affects temporal, but not spatial visual attention. Brain Cogn. 120, 8–16. doi: 10.1016/j.bandc.2017.11.004, PMID: 29222993

[ref34] Love J. Selker R. Marsman M. Jamil T. Dropmann D. Verhagen J.. (2019). JASP: graphical statistical software for common statistical designs. J. Stat. Softw. 88, 1–17. doi: 10.18637/jss.v088.i02

[ref35] Lunghi C. Sale A. (2015). A cycling lane for brain rewiring. Curr. Biol. 25, R1122–R1123. doi: 10.1016/j.cub.2015.10.026, PMID: 26654367 PMC5040496

[ref36] Lunghi C. Sframeli A. T. Lepri A. Lepri M. Lisi D. Sale A.. (2019). A new counterintuitive training for adult amblyopia. Ann. Clin. Transl. Neurol. 6, 274–284. doi: 10.1002/acn3.698, PMID: 30847360 PMC6389748

[ref37] Ma Q. (2008). Beneficial effects of moderate voluntary physical exercise and its biological mechanisms on brain health. Neurosci. Bull. 24, 265–270. doi: 10.1007/s12264-008-0402-1, PMID: 18668156 PMC5552589

[ref38] Macar F. Vidal F. Casini L. (1999). The supplementary motor area in motor and sensory timing: evidence from slow brain potential changes. Exp. Brain Res. 125, 271–280. doi: 10.1007/s002210050683, PMID: 10229018

[ref300] Marchant D. Hampson S. Finnigan L. Marrin K. Thorley C. (2020). The effects of acute moderate and high intensity exercise on memory. Front. Psychol. 11. doi: 10.3389/fpsyg.2020.01716, PMID: 32765381 PMC7381212

[ref39] Matell M. S. Meck W. H. (2004). Cortico-striatal circuits and interval timing: coincidence detection of oscillatory processes. Cogn. Brain Res. 21, 139–170. doi: 10.1016/j.cogbrainres.2004.06.012, PMID: 15464348

[ref400] Mather M. Clewett D. Sakaki M. Harley C. W. (2016). Norepinephrine ignites local hotspots of neuronal excitation: How arousal amplifies selectivity in perception and memory. Behav. Brain Sci. 39:e200. doi: 10.1017/S0140525X15000667, PMID: 26126507 PMC5830137

[ref40] Mauk M. D. Buonomano D. V. (2004). The neural basis of temporal processing. Annu. Rev. Neurosci. 27, 307–340. doi: 10.1146/annurev.neuro.27.070203.144247 15217335

[ref41] Meck W. H. (2005). Neuropsychology of timing and time perception. Brain Cogn. 58, 1–8. doi: 10.1016/j.bandc.2004.09.004, PMID: 15878722

[ref42] Merchant H. Harrington D. L. Meck W. H. (2013). Neural basis of the perception and estimation of time. Annu. Rev. Neurosci. 36, 313–336. doi: 10.1146/annurev-neuro-062012-170349, PMID: 23725000

[ref43] Niell C. M. Stryker M. P. (2010). Modulation of visual responses by behavioral state in mouse visual cortex. Neuron 65, 472–479. doi: 10.1016/j.neuron.2010.01.033, PMID: 20188652 PMC3184003

[ref44] Ohbayashi M. (2021). The roles of the cortical motor areas in sequential movements. Front. Behav. Neurosci. 15:640659. doi: 10.3389/fnbeh.2021.640659, PMID: 34177476 PMC8219877

[ref45] Petrizzo I. Anobile G. Chelli E. Arrighi R. Burr D. C. (2022). Visual duration but not Numerosity is distorted while running. Brain Sci. 12:81. doi: 10.3390/brainsci12010081, PMID: 35053824 PMC8773608

[ref46] Sansevero G. Torelli C. Mazziotti R. Consorti A. Pizzorusso T. Berardi N.. (2020). Running towards amblyopia recovery. Sci. Rep. 10:12661. doi: 10.1038/s41598-020-69630-7, PMID: 32728106 PMC7391754

[ref47] Schwarz M. A. Winkler I. Sedlmeier P. (2013). The heart beat does not make us tick: the impacts of heart rate and arousal on time perception. Atten. Percept. Psychophys. 75, 182–193. doi: 10.3758/s13414-012-0387-8 23143915

[ref48] Shiban Y. Fruth M. B. Pauli P. Kinateder M. Reichenberger J. Mühlberger A. (2016). Treatment effect on biases in size estimation in spider phobia. Biol. Psychol. 121, 146–152. doi: 10.1016/j.biopsycho.2016.03.005, PMID: 26987423

[ref49] Shibasaki H. Fukuyama H. Hanakawa T. (2004). Neural control mechanisms for normal versus Parkinsonian gait. 199–205. doi: 10.1016/S0079-6123(03)43020-3 14653165

[ref50] Singh-Manoux A. Hillsdon M. Brunner E. Marmot M. (2005). Effects of physical activity on cognitive functioning in middle age: evidence from the Whitehall II prospective cohort study. Am. J. Public Health 95, 2252–2258. doi: 10.2105/AJPH.2004.055574, PMID: 16304136 PMC1449515

[ref51] Stefanucci J. K. Storbeck J. (2009). Don’t look down: emotional arousal elevates height perception. J. Exp. Psychol. Gen. 138, 131–145. doi: 10.1037/a0014797, PMID: 19203173 PMC4712948

[ref52] Storbeck J. Stefanucci J. K. (2014). Conditions under which arousal does and does not elevate height estimates. PLoS One 9:e92024. doi: 10.1371/journal.pone.0092024, PMID: 24699393 PMC3974728

[ref500] Strath S. J. Kaminsky L. A. Ainsworth B. E. Ekelund U. Freedson P. S. Gary R. A.. (2013). Guide to the assessment of physical activity: clinical and research applications. Circulation 128, 2259–2279. doi: 10.1161/01.cir.0000435708.67487.da, PMID: 24126387

[ref53] Stryker M. P. (2014). A neural circuit that controls cortical state, plasticity, and the gain of sensory responses in mouse. Cold Spring Harb. Symp. Quant. Biol. 79, 1–9. doi: 10.1101/sqb.2014.79.024927, PMID: 25948638 PMC4500789

[ref54] Tanaka H. Monahan K. D. Seals D. R. (2001). Age-predicted maximal heart rate revisited. J. Am. Coll. Cardiol. 37, 153–156. doi: 10.1016/S0735-1097(00)01054-8, PMID: 11153730

[ref55] Terhune D. B. Russo S. Near J. Stagg C. J. Cohen Kadosh R. (2014). GABA predicts time perception. J. Neurosci. 34, 4364–4370. doi: 10.1523/JNEUROSCI.3972-13.2014, PMID: 24647956 PMC3960474

[ref56] Treisman M. (1963). Temporal discrimination and the indifference interval: implications for a model of the “internal clock”. Psychol. Monogr. Gen. Appl. 77, 1–31. doi: 10.1037/h0093864, PMID: 5877542

[ref57] van der Ham I. J. M. Klaassen F. van Schie K. Cuperus A. (2019). Elapsed time estimates in virtual reality and the physical world: the role of arousal and emotional valence. Comput. Human Behav. 94, 77–81. doi: 10.1016/j.chb.2019.01.005

[ref58] van Wassenhove V. Wittmann M. Craig A. D. B. Paulus M. P. (2011). Psychological and neural mechanisms of subjective time dilation. Front. Neurosci. 5:56. doi: 10.3389/fnins.2011.00056, PMID: 21559346 PMC3085178

[ref59] Voss M. W. Nagamatsu L. S. Liu-Ambrose T. Kramer A. F. (2011). Exercise, brain, and cognition across the life span. J. Appl. Physiol. 111, 1505–1513. doi: 10.1152/japplphysiol.00210.2011, PMID: 21527670 PMC3220305

[ref60] Voss M. W. Vivar C. Kramer A. F. van Praag H. (2013). Bridging animal and human models of exercise-induced brain plasticity. Trends Cogn. Sci. 17, 525–544. doi: 10.1016/j.tics.2013.08.001, PMID: 24029446 PMC4565723

[ref61] Waters R. P. Emerson A. J. Watt M. J. Forster G. L. Swallow J. G. Summers C. H. (2005). Stress induces rapid changes in central catecholaminergic activity in Anolis carolinensis: restraint and forced physical activity. Brain Res. Bull. 67, 210–218. doi: 10.1016/j.brainresbull.2005.06.029, PMID: 16144657

[ref62] Witt J. K. Linkenauger S. A. Bakdash J. Z. Proffitt D. R. (2008). Putting to a bigger hole: golf performance relates to perceived size. Psychon. Bull. Rev. 15, 581–585. doi: 10.3758/PBR.15.3.581, PMID: 18567258 PMC3193943

[ref63] Witt J. K. Proffitt D. R. (2005). See the ball, hit the ball. Psychol. Sci. 16, 937–938. doi: 10.1111/j.1467-9280.2005.01640.x, PMID: 16313656

[ref64] Zakay D. Block R. A. (1996). The role of attention in time estimation processes. Adv. Psychol., 143–164. doi: 10.1016/S0166-4115(96)80057-4

[ref65] Zakay D. Block R. A. (1997). Temporal cognition. Curr. Dir. Psychol. Sci. 6, 12–16. doi: 10.1111/1467-8721.ep11512604

