# Supplementary material for: Moderate physical activity alters the estimation of time, but not space
Source: Front Psychol. 2022 Oct 5;13:1004504. doi: 10.3389/fpsyg.2022.1004504 (PMC9580464; doi:10.3389/fpsyg.2022.1004504)
Supplement: Supplementary file 1 [file Data_Sheet_1.docx]

**Moderate physical activity alters the estimation of time, but not space.**

**Supplementary materials**

We run two-tails paired t-tests to see the difference in average heart rate during the three experimental sessions for each task. For both tasks we found a significant difference between the HR in the baseline and PA conditions (Distance: t = -17.88, p < 0.001, d = -4.47; Temporal: t = -17.64, p < 0.001, d = -4.41), and between the HR in the PA and POST conditions (Distance: t = 17.64, p < 0.001, d = 4.41; Temporal: t = 18.13, p < 0.001, d = 4.53). No difference in the HR was found between baseline and POST session (Distance: t = -2.32, p = 0.21, d = -0.58; Temporal: t = -1.48, p = 0.95, d = -0.37).


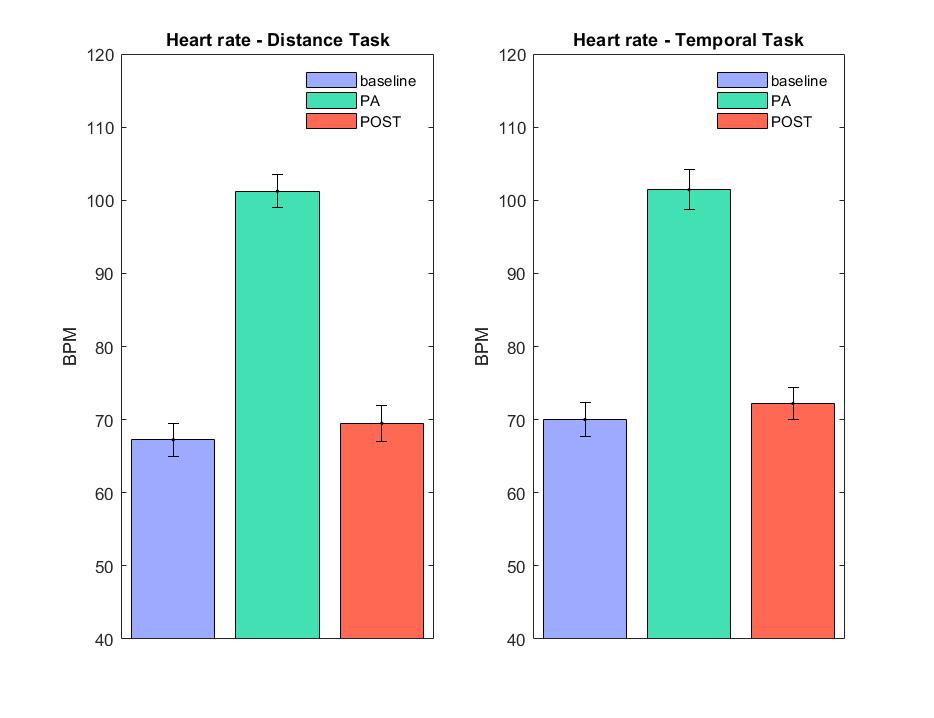


Figure 1. Average HR for each session and task.


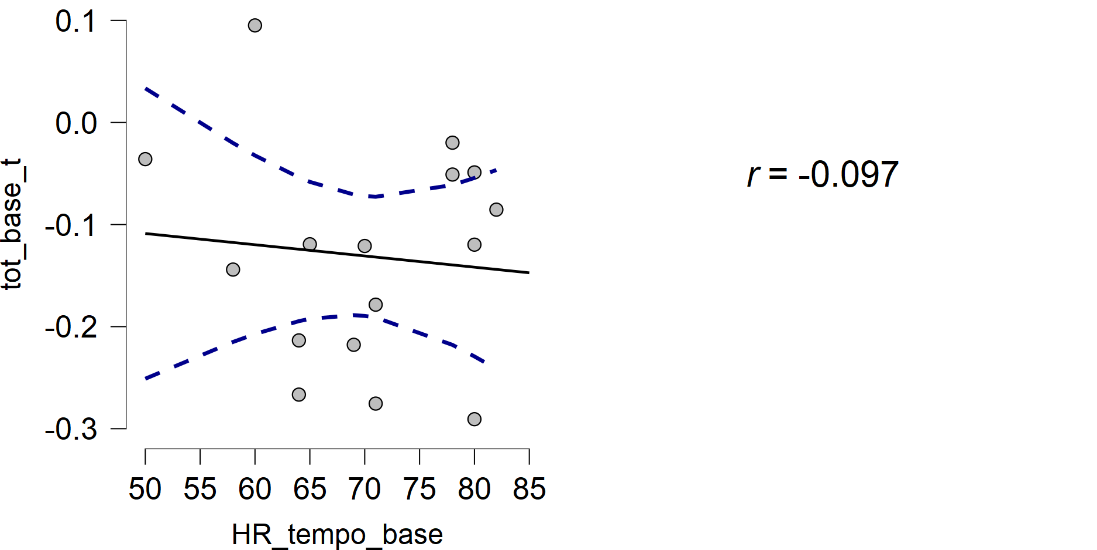

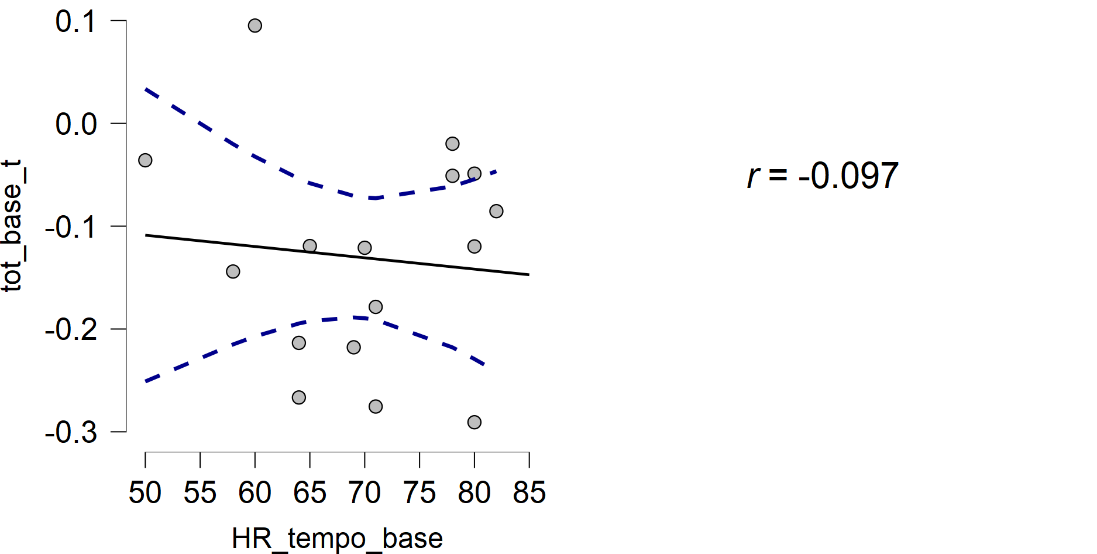


Bias Base (s)

HR base


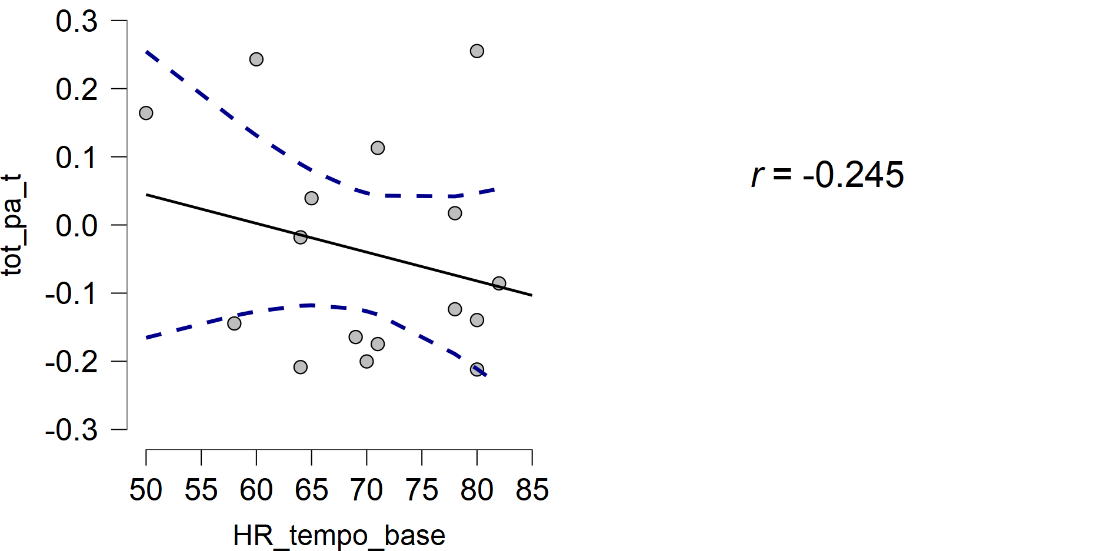

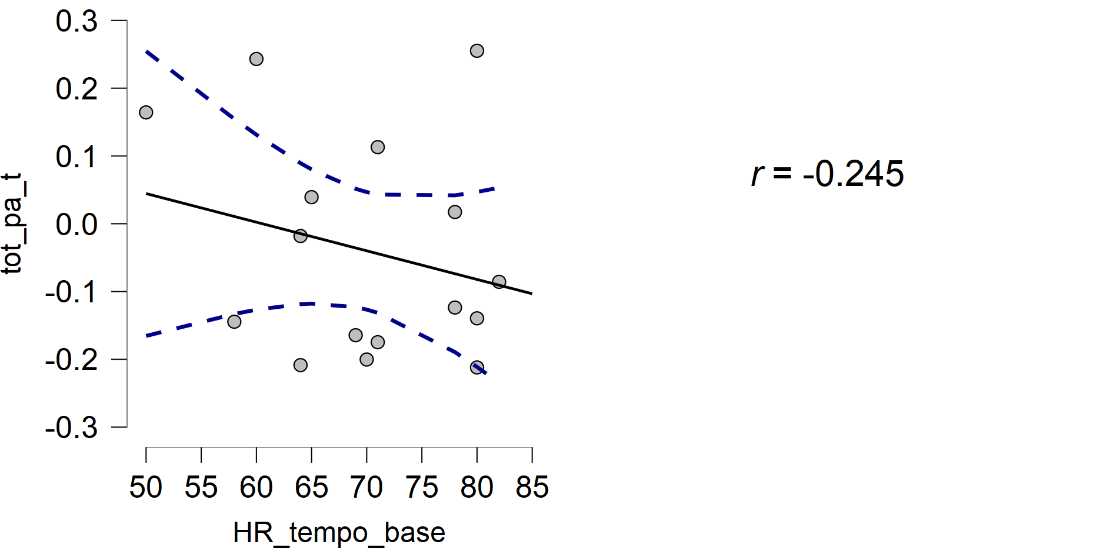


HR base

Bias PA


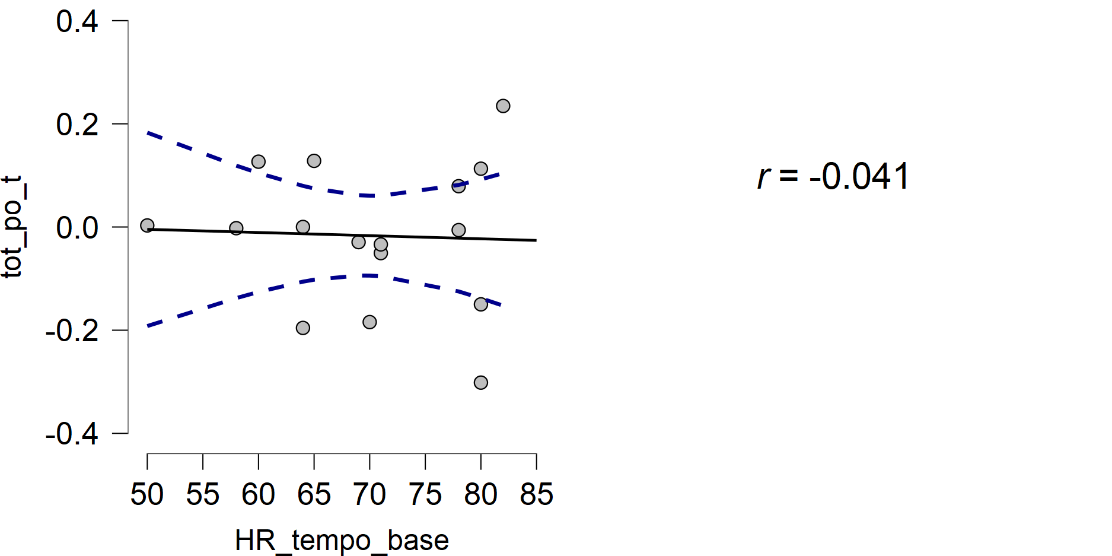

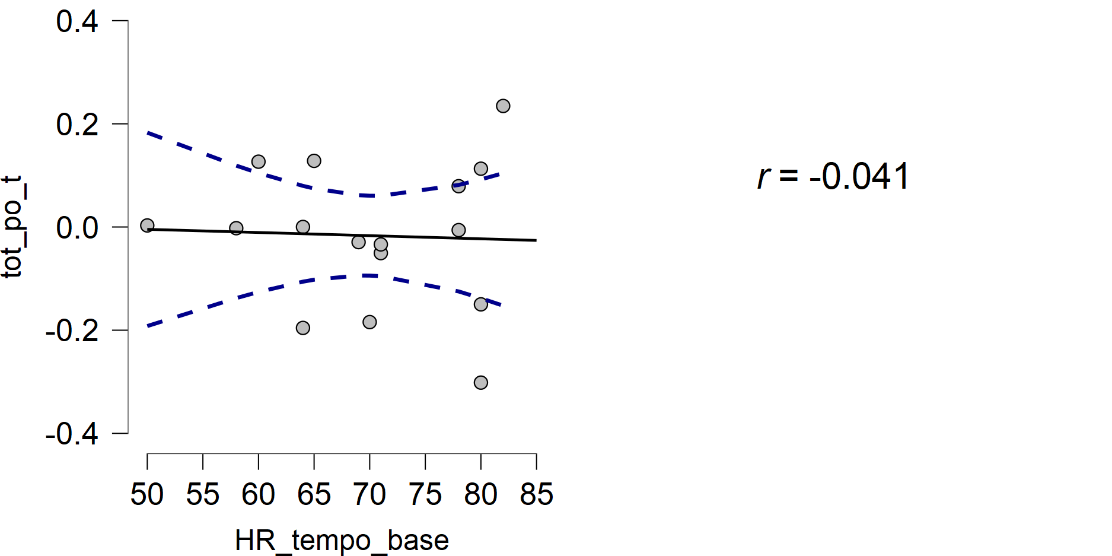


HR base

Bias POST (s)

HR base VS BIas base

HR base VS Bias PA

HR base VS Bias POST

Figure 2. Correlation between the average HR in the baseline condition and the average temporal bias for each experimental session.

To check whether the time distortion was not due to a simple change in precision, we run a Bayesian correlation analysis between the WF and the percentage of Bias in the POST session for each duration.

Bayesian correlation analysis revealed no significant correlation for none of the five duration tested (Figure 3). The Bayes factor (BF_10_) was smaller than 1 for the 200ms (BF_10_ = 0.47), 400ms (BF_10_ = 0.37) and 1600ms (BF_10_ = 0.94) in favor of the alternative hypothesis, indicating evidence in favor of the null hypothesis and rejection of the alternative hypothesis. While the duration of 800ms and 3200ms the Bayes factor was over 1 (800ms: BF_10_ = 7.02; 3200ms: BF_10_ = 2.74), but still not high enough to accept the alternative hypothesis in favor of the null.


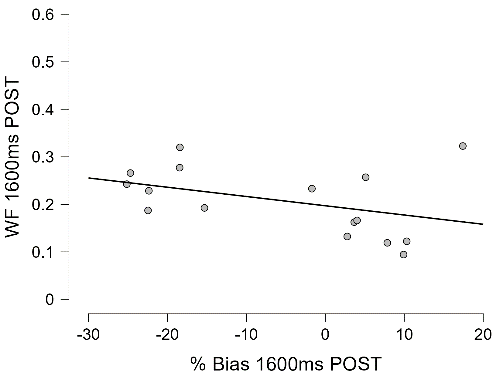

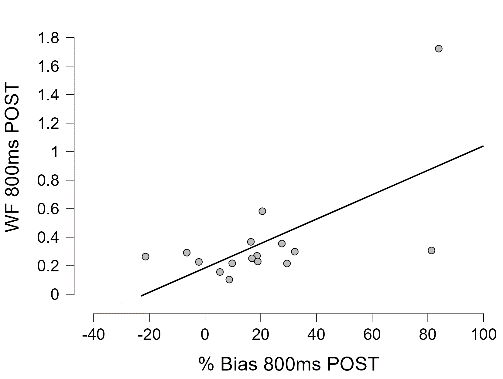

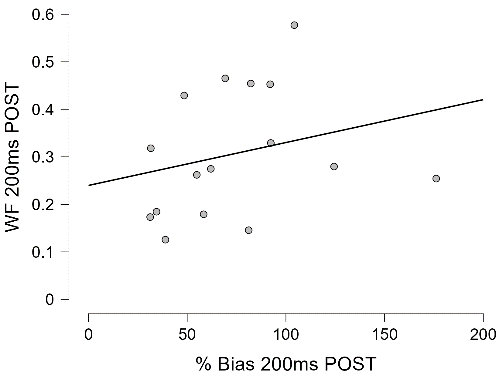

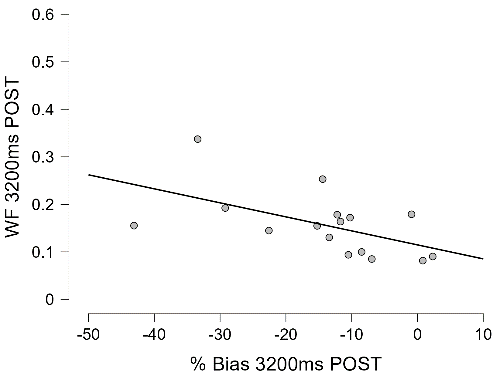

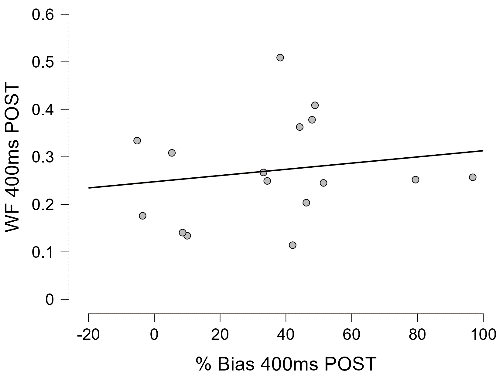


A

B

*Figure 3. A) Correlation between the average HR in the baseline condition and the average temporal bias for each experimental session*
